# Inverse pattern of GABAergic system impairment in the external versus internal globus pallidus in male heroin addicts

**DOI:** 10.1007/s00406-023-01656-0

**Published:** 2023-07-28

**Authors:** Anna Gos, Johann Steiner, Kurt Trübner, Jonas Ungewickell, Christian Mawrin, Karol Karnecki, Michał Kaliszan, Tomasz Gos

**Affiliations:** 1 Department of Psychiatry, Otto Von Guericke University, Magdeburg, Germany; 2 Institute of Legal Medicine, University of Duisburg-Essen, Essen, Germany; 3 Department of Neuropathology, Otto Von Guericke University, Magdeburg, Germany; 4 Department of Forensic Medicine, Medical University of Gdańsk, Ul. Dębowa 23, 80-204 Gdańsk, Poland

**Keywords:** Heroin addiction, Globus pallidus, GAD 65/67 immunostaining

## Abstract

Opioid addiction is a global problem that has been exacerbated in the USA and Europe by the COVID-19 pandemic. The globus pallidus (GP) plays a prominent neurobiological role in the regulation of behaviour as an output station of the striato-pallidal system. GABAergic large projection neurons are the main neuronal type in the external (EGP) and internal (IGP) parts of the GP, where addiction-specific molecular and functional abnormalities occur. In these neurons, glutamate decarboxylase (GAD) with isoforms GAD 65 and 67 is a key enzyme in GABA synthesis, and experimental studies suggest GAD dysregulation in the GP of heroin addicts. Our study, which was performed on paraffin-embedded brains from the Magdeburg Brain Bank, aimed to investigate abnormalities in the GABAergic function of large GP neurons by densitometric evaluation of their GAD 65/67-immunostained thick dendrites. The study revealed a bilaterally decreased fibres density in the EGP paralleled by the increase in the IGP in 11 male heroin addicts versus 11 healthy controls (significant U-test *P* values). The analysis of confounding variables found no interference of age, brain volume, and duration of formalin fixation with the results. Our findings suggest a dysregulation of GABAergic activity in the GP of heroin addicts, which is consistent with experimental data from animal models and plays potentially a role in the disturbed function of basal ganglia circuit in opioid addiction.

**Supplementary Information:**

The online version contains supplementary material available at 10.1007/s00406-023-01656-0.

## Introduction

The prevalence of fatal opioid overdoses has progressively increased in the United States of America since the late 1990s [1] and this increase has been significantly exacerbated by the COVID-19 pandemic [2]. Apart from illegally manufactured fentanyl, whose importance in the analysis of opioid fatalities has recently come to light, heroin remains the most important component in lethal opioid intoxications [1]. European nations have also reported similar events [3].

The striato-pallidal system is involved in subcortical-cortical loops that modulate human mood, cognitive states and behaviour, which are profoundly disturbed in addiction (for a review see: [4]). The anatomical and functional characteristics of the system allow activity to spread from the limbic to the associative and sensorimotor parts in the transfer from motivation to action [5]. Studies on habit learning, which plays a key behavioural role in addiction, support the holistic view on the system’s function [6]. The external globus pallidus (EGP) is thought to play the most important integrating and conveying role in the striato-pallidal system in primates, including humans, whereas the internal globus pallidus (IGP) is the main output structure of the system, modulating behaviour through impact on thalamocortical connectivity (for a review see: [7]).

The involvement of the limbic part of the GP located below the anterior commissure, i.e. the ventral pallidum (VP), has been highlighted mainly in animal models of addiction (for a review see: [8]). However, recent experimental studies show that drug abuse also chronically modulates the activity of projecting EGP neurons [9, 10]. In human research, both postmortem [11–13] and neuroimaging studies [14–16] revealed structural abnormalities in the GP in heroin addiction (for a review see: [17]). The neuronal population in the GP consists mainly of GABAergic projection neurons with long thick dendrites forming a dense fibre network [7, 18]. Despite morphological similarities, these neurons in the EGP and IGP have distinct neurochemical and functional properties [19, 20].

Abnormalities in the GABAergic system are thought to play a crucial role in the pathogenesis of addiction, but this is far from being elucidated (for reviews see: [4, 21]). Gamma-aminobutyric acid (GABA) is the major inhibitory neurotransmitter in the mature brain and is crucial for synaptic plasticity [22], neurogenesis [23], and synchronised oscillations of neuronal networks [24]. Experimental studies in animal models and human data suggest abnormalities in these fundamental processes in addiction, involving impaired function of GABAergic neurons [25–27]. Glutamic acid decarboxylase (GAD), with GAD 65 and 67 isoforms, is the rate-limiting enzyme involved in the conversion of glutamate to GABA. Transiently activated GAD 65 appears to be restricted to membranes and nerve terminals, while constitutively active GAD 67 is more equally distributed throughout neurons [28, 29].

Therefore, in our current study, we hypothesised that the function of GABAergic neurons in the GP is impaired in heroin-dependent individuals who died from heroin overdose. We tested this hypothesis by using GAD 65/67 immunostaining in paraffin-embedded brain sections and quantitative evaluation of the relative density of GAD 65/67-immunoreactive fibres in the GP. To assess whether the effect is distinct in both parts of the GP, we investigated bilaterally the EGP and IGP, which have different functions in the addiction-relevant circuit.

## Materials and methods

### Characteristics of the subjects

All brains were obtained from the Magdeburg Brain Bank. Sampling and preservation of the human brain material were done in accordance with the Declaration of Helsinki, German law and the local institutional review board at the University of Magdeburg. The analysis included 11 male chronic heroin addicts who died suddenly from heroin overdose and 11 male control cases of sudden natural death from cardiopulmonary arrest (see Table 2; the detailed diagnostic and demographic data of the cases examined are shown in the Supplementary Table). Information on clinical characteristics was extracted from the available clinical records and through structured interviews with persons in close contact to the heroin-dependent individuals. According to the available information, the tested heroin overdose victims exclusively met the diagnostic criteria for heroin dependence, even though they occasionally used other substances (see Supplementary Table: heroin addicts, substances used in addition to heroin). An experienced neuropathologist (C.M.) found no evidence of neurodegenerative diseases (such as Alzheimer’s, Parkinson’s, Pick’s disease), tumours, inflammatory, vascular or traumatic processes or hypoxic neuronal damage, using sections with Nissl-myelin staining and HLA-DR, beta-amyloid, and tau immunostaining to evaluate cortical regions (including the prefrontal areas), the hippocampal formation, the basal ganglia and the brainstem. None of heroin addicts was HIV-positive. A toxicological analysis of blood and urine for heroin, ethanol, and other substances of abuse was performed at each medico-legal autopsy as a part of forensic postmortem diagnostics. The cause of death in all heroin addicts was established by an experienced forensic pathologist (K.T.).

### Tissue preparation and immunohistochemistry

After embedding in paraffin, serial 20-µm thick coronal sections were cut along the rostrocaudal axis of the cerebral hemispheres and then mounted. Each 25th section was deparaffinised, rehydrated and stained with a combined cell and fibre staining according to Nissl (cresyl violet) and Heidenhain–Woelcke (myelin). Volume shrinkage was determined after dehydration and embedding of tissue using the formula: VSF = (A1/A2)^3/2^ (VSF = volume shrinkage factor, A1 = cross-sectional area before processing and A2 = cross-sectional area after processing of tissue). The median shrinkage factor was 1.99 for controls and 1.63 for heroin addicts (a non-significant *U*-test *P* value = 0.17 for the comparison between both groups).

**Fig. 1:**
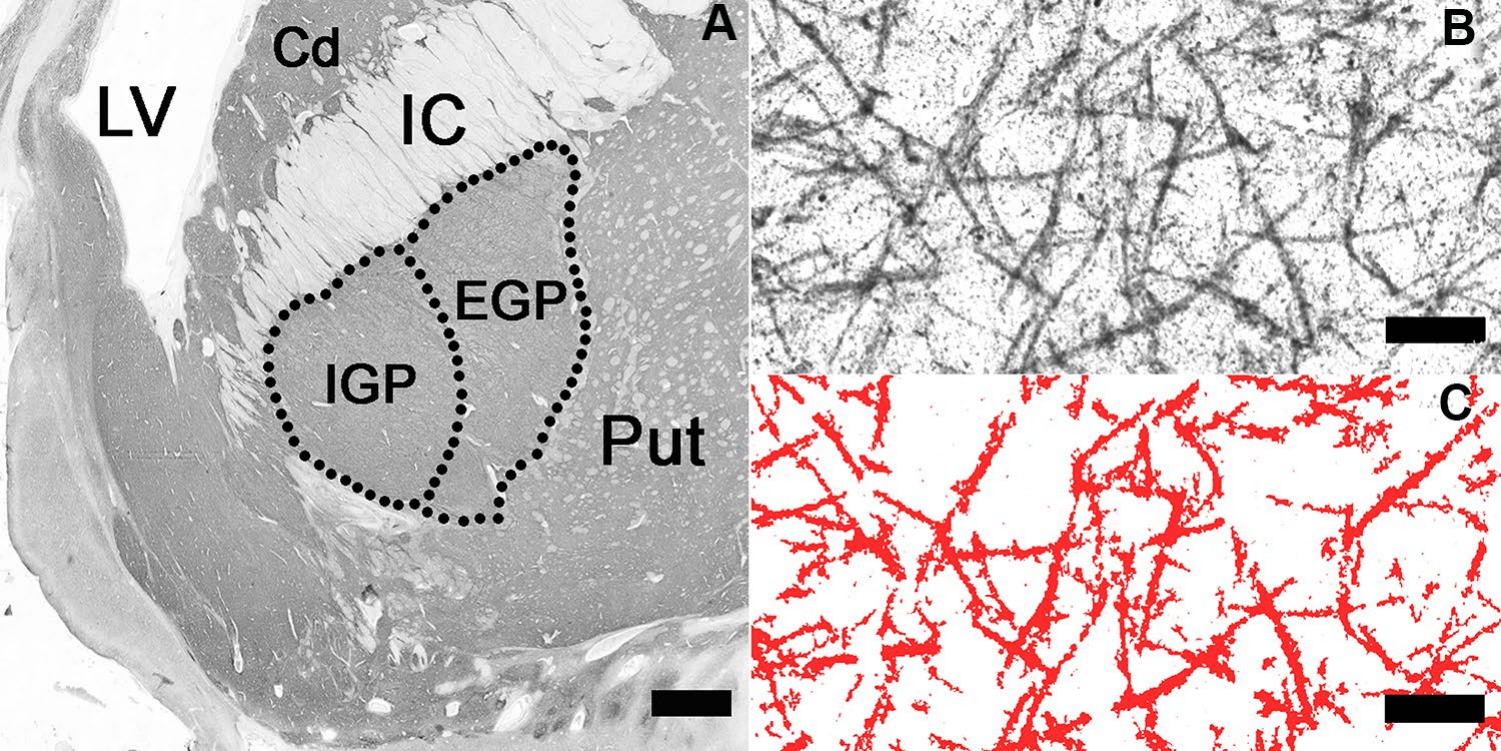
**A** Low magnification image of the fragment of the GAD 65/67-immunostained coronal section of the left brain hemisphere of the control case at the level at which the investigations were carried out (*EGP* external globus pallidus; *IGP* internal globus pallidus; *Cd* caudate nucleus; *Put* putamen; *IC* internal capsule; *LV* lateral ventricle; scale bar 2 mm). **B** Appearance of GAD 65/67-immunoreactive fibres, which was very similar in the EGP and IGP bilaterally, and in both compared groups. Long thick dendrites of GABAergic projection neurons are clearly visible and compose a dense network of fibres in both parts of the GP in accordance with previous morphological studies [18]. The differences in fibres density between heroin addicts and controls, as well as between the EGP and IGP, could not be assessed qualitatively, but could be captured by quantitative measurements (the left IGP of control case shown at **A**, scale bar 10 μm). **C** Same area as in **B**, where GAD 65/67-immunoreactive fibres marked by the CellSens computer programme were selected for automatic calculation of their relative density (scale bar 10 μm)

Stained coronal sections of the brains were taken at the level where both the EGP and IGP (clearly separated by the lamina medullaris) were visible according to established neuroanatomical criteria (Fig. 1A). From the Nissl-myelin stained sections, two were randomly selected and sections adjacent to them were stained with a mouse anti-human GAD 65/67 monoclonal antibody (Medical & Biological Laboratories Co., Woburn, USA; product number M018-3, clone 9A6, subclass mouse IgG1 kappa). The specificity of the antibody has been confirmed by the supplier using Western blotting and immunohistochemistry. Therefore, two immunostained sections separated by 0.5 mm at the level of the EGP and IGP were used to evaluate the relative GAD-ir area in these regions of interest (ROIs) bilaterally in each of the cases studied.

Formalin-fixed tissue sections were deparaffinised and antigen retrieval was performed by boiling the sections for 4 min in 10 mM citrate buffer (pH 6.0). Sections were preincubated with methanol/H_2_O_2_ to block endogenous peroxidase activity. After repeated washing with phosphate-buffered saline, the primary GAD-specific antibody was applied for 72 h at 4 °C (dilution 1:100). The washing process was repeated and sections were incubated with biotinylated anti-mouse secondary antibody (dilution 1:100) for 2 h at room temperature (Amersham Biosciences, Ltd., Little Chalfont, UK). After washing, sections were incubated with 1:100 streptavidin–biotin-peroxidase complex for 1 h at room temperature (Amersham Biosciences), 3,3′-diaminobenzidine was used for peroxidase visualisation and 0.5% ammonium nickel sulphate hexahydrate was applied to enhance the signal. Negative control sections without primary antibody showed no immunostaining.

### Quantification

Images were acquired with an Olympus BX60 microscope equipped with DP22 digital camera and CellSens Dimension Desktop v1.18 software using a 20× objective. A quantitative morphological analysis was performed in each of the selected sections as reported previously [30, 31]. The relative density of GAD-ir fibres (the quotient of the area of fibres and total measuring field area, see below) of heroin addicts and controls was measured in the EGP and IGP bilaterally. Three randomly selected boxes were approached in each of four ROIs (i.e. no specific selection criteria were applied). The number of evaluated boxes was established by the statistical analysis of previous data, where ten boxes per structure were evaluated bilaterally [30]. The area of sampling boxes was 0.340 ± 0.040 mm^2^ (this method does not require the application of a constant measuring field).

For the purpose of measuring the area of immunostained structures, the immunoreactive fibres were visualised via manual adjustments of minimum and maximum grey levels of the nickel-enhanced DAB precipitate under visual control (see Fig. 1C). Threshold adjustments were made separately for each of the sections in order to minimise the bias associated with the staining procedure. The area of the marked immunoreactive fibres was calculated and divided by the total area of the measuring field automatically, and thus demonstrated as the relative area of immunoreactive fibres (in percent). The aim was to detect the difference in GABAergic fibres density among the analysed groups according to the method described previously [30, 31] rather than obtaining absolute values of the stained fibres density.

The measurements were performed by one of the authors (A.G.) blinded to the diagnosis. In order to establish inter-rater (A.G., T.G.) and test–retest reliability repeated measurements for 5 brains were carried out. Intraclass correlation analyses yielded correlation coefficients ranging from 0.90 to 1.00 in both inter-rater and test–retest reliability evaluation.

### Data analysis

Statistical analyses were performed with the data analysis software system STATISTICA version 10 (StatSoft^®^, Inc. 2011, www.statsoft.com). As normal distribution was not given for the analysed GAD-ir fibres density (i.e. significant values of Kolmogorov and Lilliefors tests were obtained), non-parametric statistical procedures were used in hierarchical mode.

First, the generalised linear/nonlinear models (GLZ) module of STATISTICA with the general custom designs (GCD) procedure was applied as an omnibus method to analyse associations between dependent variable (i.e. GAD-ir fibres density) and independent categorical variables (i.e. heroin addiction/control groups, analysed brain region, and side). The results of GCD analysis were reported automatically including the Wald statistic value, degrees of freedom, and the respective *P* value. Age, postmortem interval, brain volume and fixation time were considered as numerical confounding variables. Spearman correlation coefficients were calculated to determine the impact of these variables which might confound GAD-ir fibres density.

Subsequently to GCD analysis, unadjusted two-way *post-hoc* comparisons with Mann–Whitney *U*-test were used to detect possible differences between the study groups with respect to the variables mentioned above (i.e. GAD-ir fibres density and confounders). All statistical tests were two-tailed. Generally, *P* values of < 0.05 were accepted as statistically significant.

## Results

### Qualitative analysis of the GAD 65/67 immunostaining

After immunostaining with the GAD-specific antibody, the dense GABAergic thick fibre systems were clearly visible in the EGP and IGP (Fig. 1B) in line with morphological studies [18]. These dense networks of GABAergic fibres in both components of the GP are composed by the long dendrites of large projection neurons, which constitute the majority of local cellular populations [18, 32], (for a review see: [7]).

### Quantitative analysis of the GAD 65/67 immunostaining

Differences in the GAD-ir thick fibres density between heroin addicts and controls could not be assessed qualitatively, but could be captured using quantitative measurements.

The omnibus analysis of results from all four investigated ROIs (i.e. from the EGP and IGP evaluated bilaterally, 44 mean values from heroin addicts and 44 mean control values) by the GCD procedure revealed strong association of diagnosis (i.e. heroin addicts vs. controls) with the investigated region (Wald statistic = 30.756, df = 1, *P* = 0.000000029). In accordance with this initial GCD analysis, further ROI-specific analyses by *post-hoc U*-tests revealed significant differences in the target parameter, which showed an inverse pattern in both regions, i.e. a decrease of GAD-ir fibres density in heroin addicts compared to controls in the EGP paralleled by an increase observed in the IGP bilaterally (see Table 1 and Supplementary Table).

**Table 1:** Results of between-group comparisons of the relative density (in percent) of glutamic acid decarboxylase immunoreactive (GAD-ir) fibres in the external and internal globus pallidus (EGP and IGP, respectively)

ROI and group	GAD-ir fibres relative density [%]	*U*-test* P*
	Median (q1, q3, n)	H/C
EGP left		0.007
H	11.10 (9.45, 13.40, 11)	
C	18.57 (12.56, 22.84, 11)	
EGP right		0.039
H	15.70 (11.23, 18.54, 11)	
C	22.72 (14.98, 31.08, 11)	
IGP left		0.004
H	20.05 (12.68, 24.99, 11)	
C	11.47 (8.72, 14.40, 11)	
IGP right		0.002
H	17.83 (15.71, 20.49, 11)	
C	11.93 (11.74, 16.00, 11)	

*ROI* region of interest; *H* heroin addicts; *C* controls; *q1 and q3* quartile 1 and 3; *n* number of cases; *U-test P* U-test *P* value

In controls (but not in heroin addicts) the target parameter was increased bilaterally in the EGP compared to the IGP (see Table 1). This may be consistent with the data on higher cellular density in the former compared to the latter part of the GP [33], considering the origin of thick fibres network [7, 18].

### Confounders

Age, brain volume and fixation time revealed significant differences between heroin addicts and controls (significant *U*-test *P* values, see Table 2 and Supplementary Table). However, correlations analysis did not suggest that the differences in GAD-ir fibres density between heroin addicts and controls observed in both GP regions were driven by these confounders (see Table 3). In particular, with regard to the accentuated difference in fixation time between the groups compared, the analysis did not suggest that longer fixation was associated with lower GAD immunoreactivity.

**Table 2:** Summary of confounding variables for heroin addicts (*n* = 11) and controls (*n* = 11). Intergroup comparisons

	BV [cm^3^]	Age [years]	PMI [hours]	Fixation [days]
Heroin addicts: median (q1, q3)	1475 (1427, 1543)	31 (25, 33)	30 (16, 49)	2922 (2185, 3815)
Controls: median (q1, q3)	1354 (1263, 1398)	46 (39, 54)	24 (24, 44)	240 (179, 330)
Statistics				
Test	*U*	*U*	*U*	*U*
Characteristic value	*Z* = − 3.021	*Z* = 2.922	*Z* = 0.263	*Z* = − 3.697
*P* value	**0.0014**	**0.0019**	0.797	**0.00002**

Significant *P* values are in bold

**Table 3:** Correlation analysis between the numerical confounding variables listed above and the relative area of glutamic acid decarboxylase immunoreactive fibres in the external and internal globus pallidus (EGP and IGP, respectively)

ROI	Group	BV	Age	PMI	Fixation
EGP left	H *r/P*	0.32/0.34	− 0.47/0.14	0.04/0.91	− 0.10/0.78
	C *r/P*	0.29/0.39	− 0.51/0.11	− 0.50/0.11	− 0.32 /0.34
EGP right	H *r/P*	0.21/0.53	0.34/0.30	0.18/0.59	**0.73/0.02**
	C *r/P*	0.55/0.08	− 0.52/0.11	0.02/0.96	− 0.27 /0.42
IGP left	H *r/P*	0.19/0.56	− 0.30/0.37	− 0.21/0.53	0.31/0.38
	C *r/P*	− 0.08/0.81	0.31/0.35	− 0.02/0.96	0.44/0.18
IGP right	H *r/P*	**0.80/0.003**	0.25/0.47	− 0.24/0.48	0.32/0.37
	C *r/P*	− 0.20/0.55	0.14/0.69	0.15/0.65	0.10/0.77

*q1 and q3* quartile 1 and 3; *BV* brain volume; *PMI* postmortem interval; *Fixation* fixation time; *ROI* region of interest; *H* heroin addicts; *C* controls; *r* correlation coefficient and *P* *P* value of the Spearman’s correlation (significant values are in bold)

## Discussion

Our study suggests an inverse pattern of GABAergic system impairment in the external versus internal globus pallidus in male heroin addicts, as we found a reduced density of GAD 65/67-ir dendrites in the EGP with a concomitant increase in the IGP. The results do not seem to be confounded by significant differences in age, brain volume and fixation time existing between the compared groups. Moreover, these opposite findings from the EGP and IGP in heroin addicts cannot be explained by the significant volume reduction in both parts of the GP found in an earlier postmortem study by Magdeburg work group [13].

**Fig. 2:**
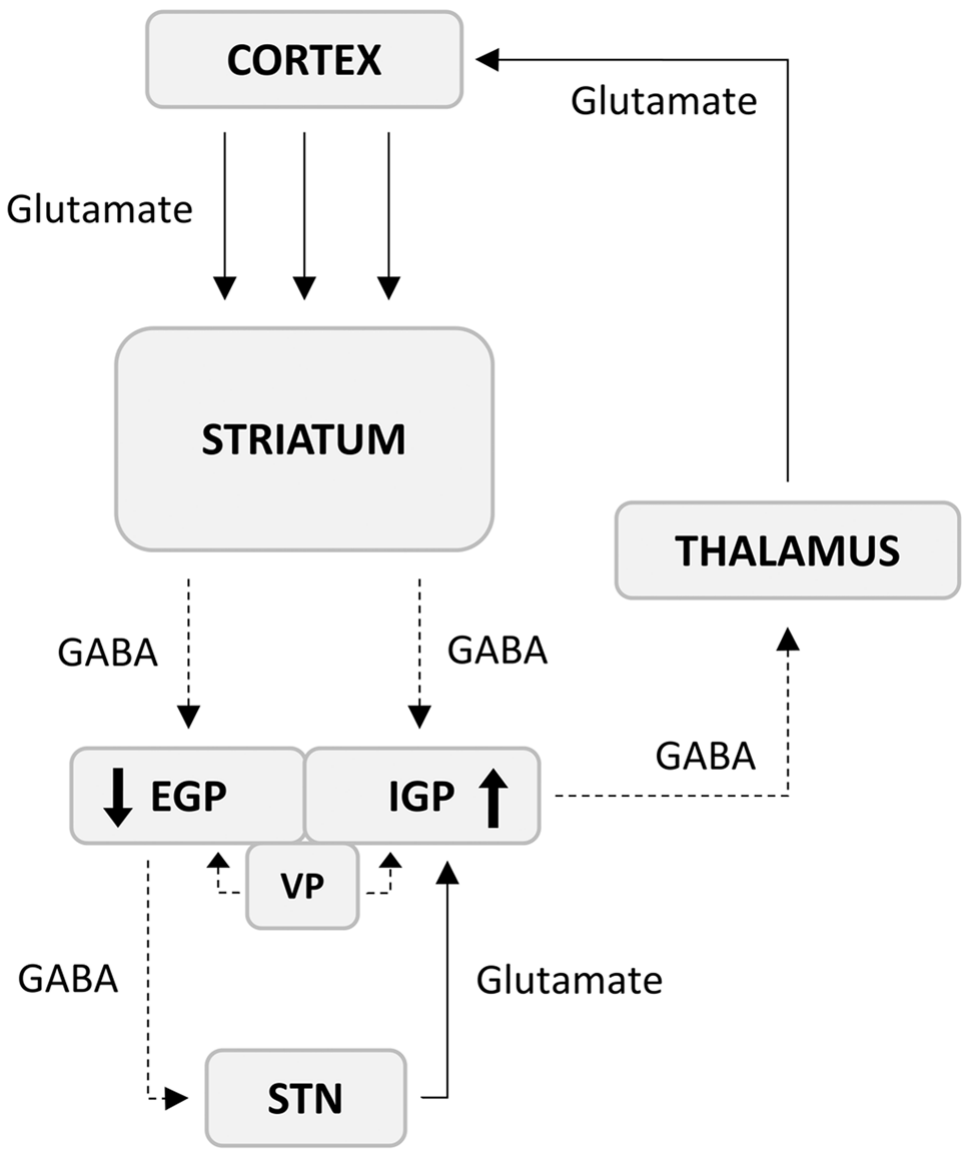
Simplified diagram of the basal ganglia circuit. Solid lines with arrows represent excitatory glutamatergic neurotransmission, and dashed lines with arrows represent GABAergic inhibitory neurotransmission (*EGP and IGP* external and internal globus pallidus; arrows in boxes correspond to decreased and increased relative density of GAD 65/67-immunoreactive fibres, respectively, observed in heroin addicts compared to controls; *VP* ventral pallidum)

The distinctness of effects observed in both parts of the GP corresponds to the different functions they perform. According to the traditional, well-established view, the EGP is a component of the indirect pathway of the basal ganglia circuit, which activates the IGP by the disinhibition of the subthalamic nucleus (see Fig. 2). On the other hand, the IGP can act independently of the EGP in the direct pathway, the activation of which leads to the opposite effect, i.e. the inhibition of the IGP [19]. Our results suggest a reduced function of the EGP, accompanied by an increased activity of the IGP, and thus the dysregulation between both pathways in heroin addiction, favouring the indirect pathway over the direct. The EGP receives GABAergic afferents from the nucleus accumbens [34], a key limbic striatal structure in the addiction circuit [4], where our recent study of the same cohort suggested increased activity of projection neurons in heroin addicts [35]. Therefore, the currently suspected decrease in EGP activity could hypothetically be related to increased inhibitory input from the nucleus accumbens.

Both the EGP and the IGP receive inputs from the VP [36], whose role in the addictive behaviour is well documented by experimental research (for a recent review see: [37]). A subpopulation of IGP neurons, together with VP neurons, project to the lateral habenular nucleus, involved in the regulation of the reward system [36, 38], where we recently found reduced volume and neuronal number in heroin addicts [39]. Therefore, the increased GABAergic activity of the IGP suggested by our results may contribute to the profound dysfunction of the reward system observed in heroin addiction [4]. In turn, EGP hypoactivity may play a role in the functional disintegration of the entire striato-pallidal system with accentuated cognitive, motivational and behavioural abnormalities seen in heroin-dependent individuals, considering the key regulatory and integrative position of the EGP in this system [7]. Our previous evaluation of rDNA transcription in large EGP neurons by the AgNOR staining suggested their reduced activity in depression, which is a frequent comorbidity in addiction [40]. In particular, EGP hypofunction may contribute to the impairment of action suspension or suppression [41], which is one of the hallmarks of addictive behaviour [4]. Accordingly, recent experimental studies in animal models have directly linked the EGP to addiction [10].

The GABAergic system is involved in many ways in the pathophysiology of addiction. GABAergic striatal outputs to the GP have been implicated in the motivation and maintenance of reinforcing behaviours towards drugs of abuse, but also in the avoidance of aversive stimuli or states [42]. After prolonged morphine administration, an increase in GAD 65/67-ir was observed in the rat GP [43], which is consistent with previous experimental data on decreased striatal GABAergic inhibitory input to the GP as a consequence of systemic or local morphine administration (for a review see: [44]).

The prominent role of the dysfunctional GABAergic system in opioid addiction is highlighted by reports on the modulation of GABAergic activity by various psychopharmacological treatments to counteract addiction-specific behavioural abnormalities. According to studies in animal models, GABA_B_ receptor modulators reduced drug seeking and reinforcing factors in opioid self-administration paradigms (for a review see: [45]). The results of experimental studies are consistent with the beneficial effects observed in the maintenance treatment of opioid dependent patients with the GABA_B_ receptor agonist baclofen, namely the attenuation of withdrawal symptoms and opioid craving [46]. In addition to GABA_B_ receptor modulation, attenuation of GABAergic activity in the subcortical addiction-relevant circuitry by nociceptin [26] and activation of dopamine D_4_ receptors [43] appear to have a therapeutic potential in opioid addiction.

GABAergic projection neurons connect cortical and subcortical structures involved in the regulation and expression of impulsivity, including the GP [47]. This behavioural endophenotype is closely related not only to addiction, but also to suicide, in which addiction plays a prominent role, according to epidemiological data [48]. D_4_ receptor activation is involved in the regulation of behavioural abnormalities in impulsive individuals [47] and restores impaired GABAergic function in the GP after prolonged morphine treatment in animal models [43]. The GABAergic system is therefore a promising target for the pharmacological treatment of addiction according to preclinical and clinical reports.

## Limitations

Our study is limited by several factors: (1) As with all postmortem analyses, our study is cross-sectional and no longitudinal data are available; (2) The cohort is small and we could only include brains from males because our brain bank does not contain postmortem brains from female heroin addicts; (3) Due to limited clinical records, there are no reliable data on the duration of addiction or the amount of heroin consumed. Therefore, we cannot assess whether these factors affect our results; (4) The use of paraffin-embedded tissue is a limitation of our method compared to frozen brain samples, which would allow the application of a wider range of approaches; and (5) Our method cannot distinguish between the populations of GABAergic neurons in the GP, which have different molecular and functional properties [49], nor between the subregions of both the EGP and IGP, which are differentially involved in the motor, associative and limbic circuits [20].

## Conclusion

In summary, our results suggest a dysregulation of GABAergic activity in the GP in heroin-dependent individuals, which may play a role in the dysfunction of the basal ganglia circuit in opioid addiction. However, further research in larger cohorts using molecular techniques targeting the different populations of GP neurons and clearly delineated subregions in both the EGP and IGP is important to substantiate these findings.

### Supplementary Information

Below is the link to the electronic supplementary material.

Supplementary file1 (DOCX 26 KB)

## Data Availability

On behalf of all authors, the corresponding author states that the data being reported are accurate and are coming from the official source.

## References

[1] Florence C, Luo F, Rice K (2021). The economic burden of opioid use disorder and fatal opioid overdose in the United States, 2017. Drug Alcohol Depend.

[2] Ahmad FB, Rossen LM, Sutton P (2023) Provisional drug overdose death counts. National Center for Health Statistics. https://www.cdc.gov/nchs/nvss/vsrr/drug-overdose-data.htm

[3] di Gaudio F, Mortali C, Tini A (2021). Opioid epidemic spread from Northern and Eastern Europe to Mediterranean Area. Clin Ter.

[4] Koob GF, Volkow ND (2016). Neurobiology of addiction: a neurocircuitry analysis. Lancet Psychiatry.

[5] Haber SN (2003). The primate basal ganglia: parallel and integrative networks. J Chem Neuroanat.

[6] Graybiel AM (2008). Habits, rituals, and the evaluative brain. Annu Rev Neurosci..

[7] Eid L, Parent M (2016). Chemical anatomy of pallidal afferents in primates. Brain Struct Funct.

[8] Root DH, Melendez RI, Zaborszky L, Napier TC (2015). The ventral pallidum: subregion-specific functional anatomy and roles in motivated behaviors. Prog Neurobiol..

[9] Abrahao KP, Chancey JH, Chan CS, Lovinger DM (2017). Ethanol-sensitive pacemaker neurons in the mouse external globus pallidus. Neuropsychopharmacology.

[10] Beier KT, Kim CK, Hoerbelt P, Hung LW, Heifets BD, DeLoach KE, Mosca TJ, Neuner S, Deisseroth K, Luo L, Malenka RC (2017). Rabies screen reveals GPe control of cocaine-triggered plasticity. Nature.

[11] Pearson J, Baden MB, Richter RW (1976). Neuronal depletion in the globus pallidus of heroin addicts. Drug Alcohol Depend.

[12] Andersen SN, Skullerud K (1999). Hypoxic/ischaemic brain damage, especially pallidal lesions, in heroin addicts. Forensic Sci Int.

[13] Müller UJ, Mawrin C, Frodl T, Dobrowolny H, Busse S, Bernstein HG, Bogerts B, Truebner K, Steiner J (2019). Reduced volumes of the external and internal globus pallidus in male heroin addicts: a postmortem study. Eur Arch Psychiatry Clin Neurosci..

[14] Al-Chalabi M, Lateef S, Gharaibeh K, Saraiya P, Ghannam M (2020). Mimicking a psychiatric disorder: heroin-induced leukoencephalopathy. Cureus..

[15] Moussawi K, Kalivas PW, Lee JW (2016). Abstinence from drug dependence after bilateral globus pallidus hypoxic-ischemic injury. Biol Psychiatry..

[16] Odia YM, Jinka M, Ziai WC (2010). Severe leukoencephalopathy following acute oxycodone intoxication. Neurocrit Care..

[17] Winstanley EL, Mahoney JJ, Castillo F, Comer SD (2021). Neurocognitive impairments and brain abnormalities resulting from opioid-related overdoses: a systematic review. Drug Alcohol Depend.

[18] François C, Percheron G, Yelnik J, Heyner S (1984). A Golgi analysis of the primate globus pallidus. I. Inconstant processes of large neurons, other neuronal types, and afferent axons. J Comp Neurol.

[19] Singh-Bains MK, Waldvogel HJ, Faull RL (2016). The role of the human globus pallidus in Huntington's disease. Brain Pathol..

[20] Saga Y, Hoshi E, Tremblay L (2017). Roles of multiple globus pallidus territories of monkeys and humans in motivation, cognition and action: an anatomical, physiological and pathophysiological review. Front Neuroanat..

[21] Koob GF (2014). Neurocircuitry of alcohol addiction: synthesis from animal models. Handb Clin Neurol.

[22] Kanold PO, Shatz CJ (2006). Subplate neurons regulate maturation of cortical inhibition and outcome of ocular dominance plasticity. Neuron.

[23] Jagasia R, Steib K, Englberger E, Herold S, Faus-Kessler T, Saxe M, Gage FH, Song H, Lie DC (2009). GABA-cAMP response element-binding protein signaling regulates maturation and survival of newly generated neurons in the adult hippocampus. J Neurosci..

[24] Gonzalez-Burgos G, Cho RY, Lewis DA (2015). Alterations in cortical network oscillations and parvalbumin neurons in schizophrenia. Biol Psychiatry.

[25] Luster BR, Cogan ES, Schmidt KT, Pati D, Pina MM, Dange K, McElligott ZA (2020). Inhibitory transmission in the bed nucleus of the stria terminalis in male and female mice following morphine withdrawal. Addict Biol.

[26] Kallupi M, Carrette LLG, Kononoff J, Solberg Woods LC, Palmer AA, Schweitzer P, George O, de Guglielmo G (2020). Nociceptin attenuates the escalation of oxycodone self-administration by normalizing CeA-GABA transmission in highly addicted rats. Proc Natl Acad Sci USA.

[27] Bayer R, Franke H, Ficker C, Richter M, Lessig R, Büttner A, Weber M (2015). Alterations of neuronal precursor cells in stages of human adult neurogenesis in heroin addicts. Drug Alcohol Depend.

[28] Laprade N, Soghomonian JJ (1999). Gene expression of the GAD67 and GAD65 isoforms of glutamate decarboxylase is differentially altered in subpopulations of striatal neurons in adult rats lesioned with 6-OHDA as neonates. Synapse.

[29] Wei J, Wu JY (2008). Post-translational regulation of L-glutamic acid decarboxylase in the brain. Neurochem Res..

[30] Gos T, Günther K, Bielau H, Dobrowolny H, Mawrin C, Trübner K, Brisch R, Steiner J, Bernstein HG, Jankowski Z, Bogerts B (2009). Suicide and depression in the quantitative analysis of glutamic acid decarboxylase-immunoreactive neuropil. J Affect Disord..

[31] Gos T, Steiner J, Bielau H, Dobrowolny H, Günther K, Mawrin C, Krzyżanowski M, Hauser R, Brisch R, Bernstein HG, Jankowski Z, Gos K, Bogerts, B,  (2012). Differences between unipolar and bipolar I depression in the quantitative analysis of glutamic acid decarboxylase-immunoreactive neuropil. Eur Arch Psychiatry Clin Neurosci..

[32] Waldvogel HJ, Billinton A, White JH, Emson PC, Faull RL (2004). Comparative cellular distribution of GABAA and GABAB receptors in the human basal ganglia: immunohistochemical colocalization of the alpha 1 subunit of the GABAA receptor, and the GABABR1 and GABABR2 receptor subunits. J Comp Neurol.

[33] Hardman CD, Henderson JM, Finkelstein DI, Horne MK, Paxinos G, Halliday GM (2002). Comparison of the basal ganglia in rats, marmosets, macaques, baboons, and humans: volume and neuronal number for the output, internal relay, and striatal modulating nuclei. J Comp Neurol.

[34] Scofield MD, Heinsbroek JA, Gipson CD, Kupchik YM, Spencer S, Smith AC, Roberts-Wolfe D, Kalivas PW (2016). The nucleus accumbens: mechanisms of addiction across drug classes reflect the importance of glutamate homeostasis. Pharmacol Rev..

[35] Gos T, Steiner J, Trübner K, Krzyżanowska M, Kaliszan M (2022). Ribosomal DNA transcription is increased in the left nucleus accumbens of heroin-dependent males. Eur Arch Psychiatry Clin Neurosci.

[36] Haber SN, Knutson B (2010). The reward circuit: linking primate anatomy and human imaging. Neuropsychopharmacology.

[37] Kupchik YM, Prasad AA (2021). Ventral pallidum cellular and pathway specificity in drug seeking. Neurosci Biobehav Rev..

[38] Hong S, Hikosaka O (2008). The globus pallidus sends reward-related signals to the lateral habenula. Neuron.

[39] Müller UJ, Ahrens M, Vasilevska V, Dobrowolny H, Schiltz K, Schlaaff K, Mawrin C, Frodl T, Bogerts B, Gos T, Truebner K, Bernstein HG, Steiner J (2021). Reduced habenular volumes and neuron numbers in male heroin addicts: a post-mortem study. Eur Arch Psychiatry Clin Neurosci..

[40] Gos T, Krell D, Bielau H, Steiner J, Trübner K, Brisch R, Bernstein HG, Jankowski Z, Bogerts B (2009). Demonstration of disturbed activity of external globus pallidus projecting neurons in depressed patients by the AgNOR staining method. J Affect Disord..

[41] Hegeman DJ, Hong ES, Hernández VM, Chan CS (2016). The external globus pallidus: progress and perspectives. Eur J Neurosci..

[42] Volkow ND, Michaelides M, Baler R (2019). The neuroscience of drug reward and addiction. Physiol Rev..

[43] Negrete-Díaz JV, Shumilov K, Real MÁ, Medina-Luque J, Valderrama-Carvajal A, Flores G, Rodríguez-Moreno A, Rivera A (2019). Pharmacological activation of dopamine D_4_ receptor modulates morphine-induced changes in the expression of GAD_65__/67_ and GABA_B_ receptors in the basal ganglia. Neuropharmacology.

[44] Xi ZX, Stein EA (2002). GABAergic mechanisms of opiate reinforcement. Alcohol Alcohol..

[45] Vlachou S, Markou A (2010). GABAB receptors in reward processes. Adv Pharmacol..

[46] Assadi SM, Radgoodarzi R, Ahmadi-Abhari SA (2003). Baclofen for maintenance treatment of opioid dependence: a randomized double-blind placebo-controlled clinical trial [ISRCTN32121581]. BMC Psychiatry.

[47] Hayes DJ, Jupp B, Sawiak SJ, Merlo E, Caprioli D, Dalley JW (2014). Brain γ-aminobutyric acid: a neglected role in impulsivity. Eur J Neurosci..

[48] Karnecki K, Gos T, Steiner J, Mańkowski D, Kaliszan M (2022). Epidemiology of suicide in the Tri-city metropolitan area in Poland in 2010–2019. Eur Arch Psychiatry Clin Neurosci.

[49] Mastro KJ, Bouchard RS, Holt HA, Gittis AH (2014). Transgenic mouse lines subdivide external segment of the globus pallidus (GPe) neurons and reveal distinct GPe output pathways. J Neurosci..

