# Supplementary material for: Inverse pattern of GABAergic system impairment in the external versus internal globus pallidus in male heroin addicts
Source: Eur Arch Psychiatry Clin Neurosci. 2023 Jul 28;274(2):445–52. doi: 10.1007/s00406-023-01656-0 (PMC10914887; doi:10.1007/s00406-023-01656-0)
Supplement: Supplementary file 1 — Supplementary file1 (DOCX 26 KB) [file 406_2023_1656_MOESM1_ESM.docx]

**Inverse pattern of GABAergic system impairment in the external versus internal globus pallidus in male heroin addicts**

Anna Gos, Johann Steiner, Kurt Trübner, Jonas Ungewickell, Christian Mawrin, Karol Karnecki, Michał Kaliszan, Tomasz Gos

**European Archives of Psychiatry and Clinical Neuroscience**

Corresponding author:

Tomasz Gos, MD, PhD

Department of Forensic Medicine

Medical University of Gdańsk

ul. Dębowa 23

80-204 Gdańsk, Poland

E-mail: [gost@gumed.edu.pl](mailto:gost@gumed.edu.pl)

**Supplementary Table** Diagnostic and demographic data, and the values of the relative density of glutamic acid decarboxylase-immunoreactive fibers in percent (GAD-ir fibers rel. density [%]) in the external and internal globus pallidus (EGP and IGP, respectively) bilaterally in controls (n=11) and heroin-dependent male subjects (n=11). *Abbreviations:* BV – brain volume; PMI – postmortem interval; Fixation – fixation time; *q1* and *q3* – quartile 1 and 3. (Significant U-test *P* values are in bold.)

| Case ID |  | BV  [cm^3^] | Age [years] | PMI [hours] | Fixation [days] | EGP left  GAD-ir fibers  rel. density [%] | EGP right  GAD-ir fibers  rel. density [%] | IGP left  GAD-ir fibers  rel. density [%] | IGP right  GAD-ir fibers  rel. density [%] |
| --- | --- | --- | --- | --- | --- | --- | --- | --- | --- |
|  | **Controls: cause of death** |  |  |  |  |  |  |  |  |
| 1 | Acute myocardial infarction | 1398.26 | 47 | 24 | 179 | 22.77 | 27.02 | 19.53 | 18.61 |
| 2 | Acute respiratory failure (aspiration) | 1157.18 | 47 | 24 | 84 | 22.84 | 14.98 | 8.72 | 16.00 |
| 3 | Sudden cardiac death | 1398.26 | 56 | 30 | 225 | 16.40 | 22.72 | 10.42 | 11.82 |
| 4 | Acute myocardial infarction | 1494.70 | 38 | 19 | 70 | 37.34 | 43.26 | 5.27 | 7.09 |
| 5 | Acute myocardial infarction | 1494.70 | 40 | 96 | 180 | 18.57 | 41.48 | 10.77 | 11.93 |
| 6 | Ruptured aortic aneurysm | 1263.26 | 64 | 35 | 240 | 9.66 | 12.15 | 13.04 | 13.01 |
| 7 | Acute respiratory failure (pneumonia) | 1353.91 | 39 | 4 | 330 | 39.76 | 18.84 | 14.40 | 13.70 |
| 8 | Pulmonary embolism (acute cor pulmonale) | 1378.98 | 54 | 24 | 250 | 10.08 | 13.65 | 12.40 | 11.74 |
| 9 | Sudden cardiac death | 1248.79 | 46 | 24 | 290 | 12.56 | 31.08 | 11.47 | 11.12 |
| 10 | Accidental strangulation (autoerotic death) | 1301.83 | 45 | 44 | 1 603 | 20.40 | 24.60 | 21.33 | 17.38 |
| 11 | Acute myocardial infarction | 1292.19 | 29 | 60 | 808 | 14.28 | 20.97 | 7.50 | 11.84 |
|  |  |  |  |  |  |  |  |  |  |
|  | *Controls: median (q1, q3)* | *1354 (1263, 1398)* | *46 (39, 54)* | *24 (24, 44)* | *240 (179, 330)* | *18.57 (12.56, 22.84)* | *22.72 (14.98,*  *31.08)* | *11.47 (8.72, 14.40)* | *11.93 (11.74, 16.00)* |
|  |  |  |  |  |  |  |  |  |  |
|  | **Heroin addicts: cause of death and** **substances used in addition to heroin** |  |  |  |  |  |  |  |  |
| 12 | Heroin overdose; alcohol, cannabis | 1407.91 | 25 | 11 | 1 372 | 9.45 | 11.23 | 12.68 | 15.71 |
| 13 | Heroin overdose; none | 1446.48 | 25 | 30 | 1 426 | 12.01 | 8.89 | 25.74 | 17.83 |
| 14 | Heroin overdose; cannabis, barbiturates, benzodiazepines, codeine | 1427.19 | 33 | 85 | 2 185 | 11.08 | 14.40 | 11.50 | 16.78 |
| 15 | Heroin overdose; cannabis | 1475.41 | 24 | 49 | 2 373 | 14.10 | 13.78 | 24.01 | 13.55 |
| 16 | Heroin overdose; alcohol, cannabis, cocaine, codeine, benzodiazepines, barbiturates | 1475.41 | 31 | 10 | 2 914 | 13.40 | 8.33 | 19.86 | 18.42 |
| 17 | Heroin overdose; alcohol, hallucinogens, cocaine, barbiturates | 1562.20 | 40 | 16 | 2 930 | 6.30 | 15.84 | 24.99 | 25.12 |
| 18 | Heroin overdose; benzodiazepines, barbiturates | 1542.91 | 32 | 16 | 3 202 | 15.39 | 24.63 | 19.87 | 24.32 |
| 19 | Heroin overdose; unknown | 1407.91 | 32 | 16 | 3 906 | 7.37 | 24.14 | 25.07 | 14.49 |
| 20 | Heroin overdose; morphine | 1446.48 | 47 | 96 | - | 10.70 | 17.49 | 11.42 | 17.46 |
| 21 | Heroin overdose; unknown | 1581.49 | 31 | 43 | 3 851 | 11.10 | 15.70 | 23.31 | 20.49 |
| 22 | Heroin overdose; unknown | 1533.27 | 21 | 32 | 3 815 | 11.48 | 18.54 | 20.05 | 18.42 |
|  |  |  |  |  |  |  |  |  |  |
|  | *Heroin addicts: median (q1, q3)* | *1475 (1427, 1543)* | *31 (25, 33)* | *30 (16, 49)* | *2922 (2185, 3815)* | *11.10 (9.45, 13.40)* | *15.70 (11.23, 18.54)* | *20.05 (12.68, 24.99)* | *17.83 (15.71, 20.49)* |
|  |  |  |  |  |  |  |  |  |  |
|  | **Statistics** |  |  |  |  |  |  |  |  |
|  | test | *U* | *U* | *U* | *U* | *U* | *U* | *U* | *U* |
|  | Characteristic value | *Z* = −3.021 | *Z* = 2.922 | *Z* = 0.263 | *Z =* −3.697 | *Z=* 2.627 | *Z* = 2.036 | *Z = −*2.758 | *Z* = −2.890 |
|  | *P* value | **0.0014** | **0.0019** | 0.797 | **0.00002** | **0.007** | **0.039** | **0.004** | **0.002** |
